# Antioxidant and Antiradical Properties of Selected Flavonoids and Phenolic Compounds

**DOI:** 10.1155/2017/7616791

**Published:** 2017-10-12

**Authors:** Zübeyir Huyut, Şükrü Beydemir, İlhami Gülçin

**Affiliations:** ^1^Department of Biochemistry, Medical Faculty, Yuzuncu Yıl University, 65080 Van, Turkey; ^2^Department of Biochemistry, Faculty of Pharmacy, Anadolu University, 26210 Eskişehir, Turkey; ^3^Department of Chemistry, Faculty of Sciences, Atatürk University, 25240 Erzurum, Turkey

## Abstract

Phenolic compounds and flavonoids are known by their antioxidant properties and one of the most important sources for humans is the diet. Due to the harmful effects of synthetic antioxidants such as BHA and BHT, natural novel antioxidants have become the focus of attention for protecting foods and beverages and reducing oxidative stress* in vivo*. In the current study, we investigated the total antioxidant, metal chelating, Fe^3+^ and Cu^2+^ reduction, and free radical scavenging activities of some phenolic and flavonoid compounds including malvin, oenin, ID-8, silychristin, callistephin, pelargonin, 3,4-dihydroxy-5-methoxybenzoic acid, 2,4,6-trihydroxybenzaldehyde, and arachidonoyl dopamine. The antioxidant properties of these compounds at different concentrations (10–30 *μ*g/mL) were compared with those of reference antioxidants such as BHA, BHT, *α*-tocopherol, and trolox. Each substance showed dose-dependent antioxidant activity. Furthermore, oenin, malvin, arachidonoyl dopamine, callistephin, silychristin, and 3,4-dihydroxy-5-methoxybenzoic acid exhibited more effective antioxidant activity than that observed for the reference antioxidants. These results suggest that these novel compounds may function to protect foods and medicines and to reduce oxidative stress* in vivo*.

## 1. Introduction

Reactive oxygen species (ROS) are continuously formed by normal cellular processes endogenously and environmental factors exogenously [1]. ROS include nonradical species such as hydrogen peroxide (H_2_O_2_), hypochlorous acid (HOCl), singlet oxygen (^1^O), and free radicals such as superoxide anion radical (O_2_^∙−^), hydroxyl radical (OH^∙^), and hydroperoxide (ROO^∙^) [2–4]. Free radicals at physiological concentrations have a series of useful biological functions such as acting as a cell signaling molecule; functioning against cellular responses; controlling cell viability, migration, and differentiation; protecting cells against pathological and infectious agents and inactivating them [5–7]. However, levels of ROS higher than physiological concentrations cause oxidative-antioxidant imbalance and oxidative stress [8]. Oxidative stress is a factor that induces a number of diseases such as atherosclerosis, cardiovascular diseases, diabetes, inflammation, aging, skin lesions, rheumatoid arthritis, and neurological diseases [9–11]. When enzymatic or nonenzymatic endogenous antioxidants are inadequate to remove ROS from the body, it becomes important for the body to receive exogenous natural antioxidants such as phenolic compounds.

Phenolic compounds are secondary plant metabolites that are found naturally in all plant materials, including plant-based food products [12]. These compounds are thought to be an integral part of human and animal diets. They represent the most important group of natural antioxidants [13]. The most common phenolic compounds in plants can be classified into phenolic acids, tocopherols, and flavonoids [14]. It has been reported that phenolic and flavonoid compounds act as antioxidants to exert antiallergic, anti-inflammatory, antidiabetic, antimicrobial, antipathogenic, antiviral, antithrombotic, and vasodilatory effects and prevent diseases such as cancer, heart problems, cataracts, eye disorders, and Alzheimer's [15–17]. In addition, the most important features of flavonoids include their ability to protect against oxidative diseases, activate or inhibit various enzymes bind specific receptors, and protect against cardiovascular diseases by reducing the oxidation of low-density lipoproteins [18].

Various methods have been developed to investigate the antioxidant properties of a substance. These include assays for total antioxidant capacity (TAC), NO^∙^, H_2_O_2_, O_2_^∙−^, and OH^*∙*^ radical scavenging capacity; oxygen radical scavenging activity (ORAC); Fe^3+^ and Cu^2+^ reducing activity (FRAP and CUPRAC assay, resp.); metal chelating activity; ABTS^∙+^, DMPD^∙+^, and DPPH^∙^ free radical scavenging activity; and lipid peroxidation inhibition capacity [19–21]. Among these, the most commonly used methods are TAC determination and assays for determining Fe^+3^ and Cu^+2^ reduction activity, metal chelating ability, and free radical (ABTS^∙+^, DPPH^∙^, DMPD^∙+^, OH^∙^, and O_2_^∙−^) scavenging activity [21].

**Figure 1:**
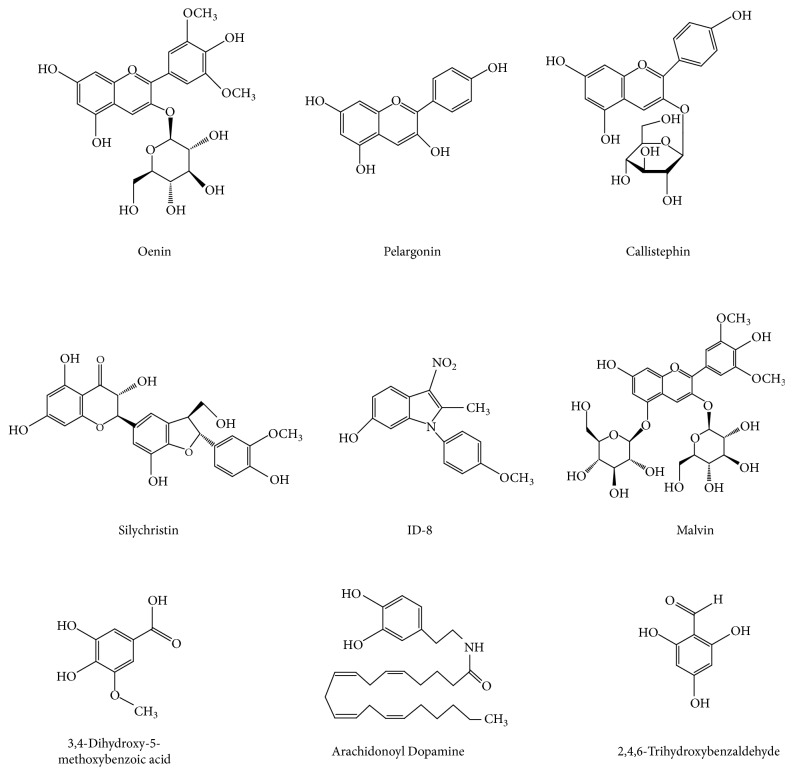
Molecular structures of flavonoid and phenolic substances used as antioxidant in this study.

In the current study, we investigated the antioxidant capacities of malvin, oenin, ID-8, silychristin, callistephin, and pelargonin with flavonoid structures and 3,4-dihydroxy-5-methoxybenzoic acid, arachidonoyl dopamine, and 2,4,6-trihydroxybenzaldehyde with phenolic structures (Figure 1) by assaying, Fe^3+^ and Cu^2+^ reduction activity, metal chelating activity, and O_2_^∙−^, ABTS^∙+^, DPPH^∙^, and DMPD^∙+^ radical scavenging capacity.

## 2. Materials and Methods

### 2.1. Chemicals

Sodium dihydrogen phosphate (NaH_2_PO_4_), potassium ferrocyanide (K_3_Fe(CN)_6_), trichloroacetic acid (TCA), iron-III-chloride (FeCl_3_), potassium peroxydisulfate (K_2_O_8_S_2_) copper-II-chloride (CuCl_2_), sodium acetate (NaCH_3_COO), hydrochloric acid (HCl), tris, iron-II-sulfate (FeSO_4_), iron-II-chloride (FeCl_2_), disodium hydrogen phosphate (Na_2_HPO_4_), methionine, ethanol, ethylenediaminetetraacetic acid (EDTA), ammonium thiocyanate (NH_4_SCN), sodium bicarbonate (NaHCO_3_), sodium hydroxide (NaOH), disodium sulfate (Na_2_SO_4_), sodium perchlorate (NaClO_4_), and disodium carbonate (Na_2_CO_3_) were obtained from Merck (Merck, made in Germany). Cis-9, cis-12-octadecanoic acid (linoleic acid), 2,2-diphenyl-1-picrylhydrazyl (DPPH), polyoxyethylene sorbitan monolaurate (Tween 20), nitroblue tetrazolium (NBT), 3-(2-pyridyl)-5,6-diphenyl-1,2,4-triazine-4′,4′′-disulfonic acid sodium salt (ferrozine), riboflavin (vitamin B2), N-N-Dimethyl-p-phenyl-enediamine dihydrochloride (DMPD), neocuproine hydrate (C_4_H_12_N_2_·*X*H_2_O), 2,2′-azino-bis(3-ethylbenzothiazoline-6-sulfonic acid) diammonium sulfate (ABTS), ID-8 (C_6_H_14_N_2_O_4_), 3,4-dihydroxy-5-methoxybenzoic acid (C_8_H_8_O_5_), silychristin (C_25_H_22_O_10_), malvin (C_29_H_28_O_17_), pelargonin (C_27_H_31_O_15_), oenin (C_23_H_25_O_2_), arachidonoyl dopamine (C_28_H_41_NO_3_), callistephin (C_21_H_21_O_10_), and 2,4,6-trihydroxy-benzaldehyde (C_6_H_2_CHO) were purchased from Sigma Aldrich Company (St Louis, MO, USA).

### 2.2. Determination of TAC

TAC was determined by the thiocyanate method [22]. Different concentrations of the stock solutions (10, 20, 30 *μ*g/mL) of phenolic and flavonoid compounds were added to tubes and volume was brought to 2.5 mL using phosphate buffer (0.01 M and pH 7.4). Subsequently, 2.5 mL of linoleic acid emulsion was added to each tube, and the mixture was incubated at 37°C in the dark. Samples (100 *μ*L) were taken every 12 h during incubation, to which 4.7 mL of ethanol, 100 *μ*L of SCN^−^ solution, and 100 *μ*L of Fe^2+^ solution (20 mM) prepared in HCl (3.5%) were added. The absorbance of the samples at 500 nm was measured and compared to that of blank solution. Alcohol was used instead of the sample for the blank, while buffer solution was used instead of the sample for the control. The incubation and absorbance measurements were continued until the maximum absorbance values of the control sample were reached (about 1.5 d).

### 2.3. Determination of Fe^3+^-Fe^2+^ Reduction Activity by FRAP Reagent (FRAP Assay)

The reduction power as FRAP reactivity was determined using the method of Oyaizu with slight modification [23]. Different solutions (10, 20, 30 *μ*g/mL) were prepared from the 1 mg/mL stock solutions of the phenolic and flavonoid compounds. The sample volume in the tubes was to 0.5 mL using acetate buffer (pH 3.6). Subsequently, 2.25 mL of FeCl_3_ solution and 2.25 mL of FRAP reagent were added to each tube (total volume 5 mL). After incubation for 10 min, the absorbance of the mixture was read at 593 nm against the blank. Acetate buffer was used as a blank control sample.

### 2.4. Cu^2+^-Cu^+^ Reduction Activity (CUPRAC Assay)

The Cu^2+^ reduction activity was determined using the method previously described by Apak et al. with a minor modification [24]. Different concentrations (10, 20, and 30 *μ*g/mL) of phenolic and flavonoid compounds were mixed with 125 *μ*L of CuCl_2_ solution (0.01 M), 125 *μ*L of ethanolic neocuprin solution (7.5 mM), and 125 *μ*L of ammonium acetate buffer solution (1 M). After incubation in the dark for 30 min, absorbance was measured at 450 nm against a distilled water blank.

### 2.5. Superoxide Anion Radical (O_2_^∙−^) Scavenging Activity

Superoxide anion radical scavenging activity was determined using the method described by Zhishen et al. with slight modification [25]. This method is based on the spectrophotometric measurement of nitroblue tetrazolium (NBT). Different concentrations of samples and standards were prepared in phosphate buffer (0.05 M and pH 7.8). To the sample solutions, riboflavin, methionine, and NBT were added at concentrations of 13.3, 44.6, and 81.5 × 10^−2^ *μ*M, respectively. The reaction mixture was stimulated with 20 W of fluorescent light at room temperature for 2 h. Absorbance was measured at 560 nm against a distilled water blank.

### 2.6. DPPH Radical Scavenging Activity

The DPPH radical scavenging activity was analyzed according to the method of Blois [26]. DPPH solution (1 mM) was used as the free radical. The previously prepared 1 mg/mL antioxidant stock solutions were used. Samples were added to test tubes at concentrations of 10, 20, and 30 *μ*g/mL and the total volume was brought to 2.5 mL using pure ethanol. Subsequently, 0.5 mL of the stock DPPH solution was added to each sample tube. After incubation at room temperature in the dark for 30 min, the absorbance values were measured at 517 nm against the ethanol blank. A solution of 2 mL of ethanol and 0.5 mL of DPPH solution was used as a control. Decreasing absorbance values indicated higher free radical scavenging activity.

### 2.7. ABTS Radical Scavenging Activity

ABTS radical elimination activity was measured using the method of Re et al. [27]. First, ABTS solution (2 mM) was prepared in phosphate buffer (1 M and pH 7.4). ABTS radicals were produced by adding 2.45 nM persulfate solution to the mixture. Next, the absorbance of the control solution at 734 nM was adjusted to 700 ± 0.025 nm using phosphate buffer (0.1 M and pH 7.4). ABTS radical solution (0.5 mL) was added to different concentrations (10–30 *μ*g/mL) of the antioxidants used in this study and incubated for 30 min. The absorbance was measured against an ethanol blank at 734 nm.

### 2.8. DMPD Radical Scavenging Activity

For this assay, a colored radical cation (DMPD^*∙ ***+**^) was first obtained. For this purpose, 1 mL of DMPD solution and 0.2 mL of 0.05 M FeCl_3_ were added to 100 mL of acetate buffer (pH 5.3; 100 mM), thus forming the DPPH radical solution. The optical density of the control solution at 505 nm was adjusted to 0.900 ± 0.100 nm using phosphate buffer (0.1 M and pH 5.3). The absorbance of freshly prepared DMPD^∙+^ solution is stable for 12 h. Different concentrations (10–30 *μ*g/mL) of some phenolic and flavonoid compounds and reference antioxidants were transferred to the test tubes and the total volume was brought to 0.5 mL using distilled water. One milliliter of DMPD^∙+^ solution was added to the solution and absorbance values were measured at 505 nm after incubation for 50 min. Buffer solution was used as a blank [28].

### 2.9. Fe^2+^ Chelating Activity

Metal chelating activities of the phenolic and flavonoid compounds and positive control substances were assay using the method previously described by Dinis et al. [29]. The phenolic and flavonoid compounds were added at different concentrations (10, 20, and 30 *μ*g/mL) to a solution containing 50 *μ*L of FeCl_2_·4H_2_O (2 M) and 350 *μ*L of purified water. The final volume was brought to 4 mL using distilled water. The reaction was initiated by adding 0.2 mL of ferrozine solution (5 mM). After the solution was thoroughly mixed by vortexing, it was incubated at room temperature for 10 min. Subsequently, the absorbance values were measured at 562 nm against an ethanol blank. As a control, a solution lacking any phenolic or flavonoid compounds was used.

## 3. Results and Discussion

Antioxidant compounds exert their effects through different mechanisms such as inhibiting hydrogen abstraction, binding transition metal ions, radical scavenging, and disintegrating peroxides [30, 31]. One of the most important factors influencing antioxidant capacity is the ability of the antioxidant to donate electrons. Due to the harmful effects of synthetic antioxidants such as BHA and BHT, antioxidant capacities of flavonoids and phenolic compounds in plant-derived or natural origin have garnered substantial research interest and are being investigated extensively [32]. Many methods have been developed to determine the antioxidant capacities of synthetic or naturally sourced compounds, plant extracts, and other samples. Among these methods, TAC; reducing power, DPPH, DMPD, ABTS^*∙*+^, and O_2_^∙−^ scavenging ability; and metal chelating activities are the most frequently used [21].

TAC determination is a method that encompasses many factors, which are captured individually by other methods. Since TAC is affected by metal chelating capacity, reducing power, and free radical scavenging activity of compounds (e.g., by the number of -OH groups bound to aromatic rings and conjugate diene structure of antioxidant molecules), it is obvious that each method should be applied and evaluated separately [33].

TAC determination is widely used for clinically used bioactive substances and compounds that are food ingredients. TAC can also be defined as the capacity to inhibit lipid peroxidation of compounds [34]. The ability to inhibit linoleic acid emulsion is tested to determine possible total antioxidant effects of a bioactive compound [35]. Linoleic acid emulsion ultimately produces hydroperoxides and the resulting hydroperoxides decompose to form secondary products. In this method, the amount of hydroperoxide from the linoleic acid resulting from autoxidation is measured indirectly during the test period. Hydroperoxides react with Fe^2+^ to form Fe^3+^. These secondary ions (Fe^3+^) form complexes with thiocyanate (SCN^−^). The resulting Fe(CN)^2+^ complex exhibits a maximum absorbance at 500 nm. The oxidation of linoleic acid is slow in the presence of antioxidants [36]. Therefore, the greater the ability to inhibit the oxidation of Fe^2+^ to Fe^3+^ of the antioxidant substance, the lower the absorbance will be. In this study, the thiocyanate method was used to determine the TAC of a reference antioxidant and various phenolic and flavonoid compounds: their ability to inhibit linoleic acid emulsion at a 20 *μ*g/mL concentration was determined. ID-8, callistephin, malvin, and oenin had higher inhibitory effects than all reference antioxidants used, with 97.98%, 98.90%, 96.75%, and 96.7% inhibition values, respectively, at 36th h (Table 1).

In addition, malvin, pelargonin, and silychristin exhibited inhibition values of 95.16%, 93.93%, and 95.45%, respectively, showing better lipid peroxidation inhibitory activity than the reference antioxidants BHT, *α*-tocopherol, and trolox. When the TACs of the reference antioxidants and the phenolic and flavonoid compounds were compared, the antioxidant activity observed, from highest to lowest, was as follows: ID-8 > callistephin > oenin > BHA > silychristin > malvin > pelargonin > trolox > BHT > arachidonoyl dopamine > 2,4,6-trihydroxybenzaldehyde > 3,4-dihydroxy-5-methoxybenzoic acid > *α*-tocopherol.

Elemental species such as Fe^2+^ accelerate ROS production in the body. Therefore, the Fe chelating activity of a substance may be related to its antioxidant activity. Among transition metals, Fe is known as the most important prooxidant that causes lipid peroxidation due to its high reactivity. Effective Fe^2+^ ion chelators prevent oxidative damage and oxidative stress-based diseases by binding Fe^2+^ ions, which can produce OH^*∙*^ radicals and are very reactive in Fenton-type reactions [37].

Similarly, this method is also performed using bipyridyl reactives. With this method, 3,4-dihydroxy-5-methoxybenzoic acid with 92% metal chelating capacity at 10 *μ*g/mL concentration was more effective than the reference antioxidants and other phenolic and flavonoid compounds did, with the exception of EDTA (95.80%). In addition, ID-8 and arachidonoyl dopamine with 88.06% and 73.86% metal chelating activity, respectively, demonstrated higher metal chelating activity than the other phenolic and flavonoid compounds and reference antioxidants did, with exception of EDTA and *α*-tocopherol. Reference antioxidants and some phenolic and flavonoid compounds exhibited metal chelating activity to varying degrees (Table 1).

In the presence of chelating agents, the red color of the Fe^2+^-ferrozin complex, which exhibits maximum absorbance at 562 nm as a result of the reduction, decreases. Measuring the color decrease provides an estimate of the metal chelating activity of the chelating agent. Low absorbance indicates high metal chelating activity [38]. Kazazica et al. reported that flavonoids such as campherol exhibit Cu^2+^ and Fe^2+^ chelating activity via their functional groups [39]. Similarly, Fiorucci et al. showed that quercetin exhibits metal ion binding activity [40]. In another study, it was determined that L-carnitine chelates Fe^2+^ ions via its carbonyl and hydroxyl functional groups. Likewise, it has been proposed that curcumin chelates ferrous ions via its carbonyl and hydroxyl functional groups [41]. Similarly, L-adrenaline binds iron ions via its amine and hydroxyl groups [42]. We tested metal chelating activities of reference antioxidants and selected phenolic and flavonoid compounds at different concentrations (10–30 *μ*g/mL) using ferrozine and bipyridyl reagents. In our study, ID-8, malvin, arachidonoyl dopamine, and pelargonin exhibited higher Fe^2+^ chelating activity than reference antioxidants and other phenolic and flavonoid compounds did at 10 *μ*g/mL by chelating metal ions at levels of 54.16%, 52.21%, 50.65%, and 39.58%, respectively (Table 1). In addition, ID-8, arachidonoyl dopamine, malvin, and pelargonin, with IC_50_ values of 18.33, 18.54, 25.72, and 20.37 *μ*g/mL, respectively, exhibited more effective Fe^2+^ ion chelating activity than reference antioxidants and the other phenolic and flavonoid compounds tested did (Table 1). Additionally, we hypothesized that Fe^2+^ chelating activities of the compounds in this study may be due to their -OH groups.

Determining metal chelating activity using the bipyridyl reagent was performed at different concentrations (10–30 *μ*g/mL) of reference antioxidants and selected phenolic and flavonoid compounds. In the absorbance-quantity plot drawn according to the results obtained using bipyridyl reagent (Table 1), IC_50_ values of each substance were calculated from the curve corresponding to the 10 *μ*g/mL concentration. ID-8 and arachidonoyl dopamine exhibited better metal chelating activity than other phenolic and flavonoid compounds tested and reference antioxidants did, except EDTA, with IC_50_ values of 8.80 and 11.08 *μ*g/mL, respectively (Table 1).

Free radical scavenging activity is very important because of the harmful effects of free radicals in biological systems and foods. Radical scavengers can react with free radicals directly to clear peroxide radicals, enhance the stability and quality of food products and drugs, and terminate peroxidation chain reactions [43]. This test is one of the standard tests in antioxidant activity studies and provides rapid results for the radical scavenging activity of specific compounds [44]. Free radicals scavenging assays based on the scavenging of DPPH^*∙*^, DMPD^*∙*+^, ABTS^*∙*+^, and O_2_^∙−^ radicals are the most popular spectrophotometric methods used to determine the antioxidant capacities of foods, beverages, and plant extracts. In addition, these have advantages such as inexpensive reagents, less labor requirements, ease of use, high sensitivity, and ability to rapidly analyze antioxidant properties of numerous samples without complicated instruments [45]. When antioxidants are added to a medium containing radicals, DPPH^∙^, DMPD^∙+^, and ABTS^∙+^ radicals are converted into their reduced forms, resulting in decolorization of the solution.

The DPPH radical scavenging assay is one of the oldest methods for determination of antioxidant activity [34]. The DPPH radical is an unstable organic nitrogen radical with a dark blue color. In this method, antioxidants reduce the stable DPPH radicals to yellow diphenyl-picrylhydrazine. This method is based on the fact that these radicals are converted to DPPH-H, the nonradical reduced form of the DPPH radicals, upon hydrogen donation by antioxidants in the alcohol solution [46]. The purple-colored, stable, free DPPH radical exhibits maximum absorbance at 517 nm. When DPPH radicals contact a proton donor substrate, they are cleared and the absorbance decreases [47].

Resveratrol is one of the main phenolic compounds found in grapes. Gülçin showed that resveratrol is an effective DPPH radical scavenger [48]. The DPPH^*∙*^ scavenging activity of the reference antioxidants and phenolic and flavonoid compounds at different concentrations (10–30 *μ*g/mL) was measured at 517 nm. As the concentration of the substance increased the amount of free radicals in the mixture decreased proportionally for almost all phenolic and flavonoid compounds. In our study, 3,4-dihydroxy-5-methoxybenzoic acid, with an IC_50_ value of 10.69 *μ*g/mL showed more DPPH radical scavenging activity than the reference antioxidants BHT, *α*-tocopherol, and trolox. ID-8 with an IC_50_ value of 536.41 *μ*g/mL exhibited the lowest DPPH radical scavenging activity of the compounds examined. However, all the test materials showed dose-dependent DPPH radical scavenging activity (Table 2).

Superoxide anion radicals are biologically highly toxic and are produced by the immune system to kill microorganisms.* In vivo*, superoxide can be produced as a result of an electron being transferred to oxygen because of various metabolic processes or activation of oxygen by a radical [49]. Although superoxide radicals have relatively limited chemical reactivity and are a weak oxidant, they can produce very dangerous reactive components such as singlet oxygen and hydroxyl radicals that cause lipid peroxidation [50]. It has also been observed that superoxide radical directly initiates lipid peroxidation [51]. When the riboflavin used in this method is photochemically activated, it reacts with NBT to produce NBTH^*∙*^. The NBTH radical leads to formazan formation. In the presence of oxygen, radical species are controlled by a semiequilibrium reaction. With the presence of antioxidants that donate electrons to NBT, the degradation of the typical purple color of formazan can be monitored spectrophotometrically at 560 nm. Antioxidants have the ability to inhibit the conversion of NBT. Decreased absorbance at 560 nm in the presence of antioxidants indicates that the superoxide anion radicals are scavenged [9]. The results obtained with this method showed that 3,4-dihydroxy-5-methoxybenzoic acid and pelargonin, with IC_50_ values of 11.47 and 14.13 *μ*g/mL, respectively, possessed better O_2_^∙−^ anion radical scavenging activity than the other phenolic and flavonoid compounds and the reference antioxidants BHA, BHT, *α*-tocopherol, and trolox did (Table 2). Additionally, oenin, callistephin, silychristin, and 2,4,6-trihydroxybenzaldehyde showed better O_2_^∙−^ anion radical scavenging properties than the reference antioxidants BHA, *α*-tocopherol, and trolox did, and all other substances exhibited dose-dependent O_2_^∙−^ scavenging activity.

ABTS is oxidized by oxidants into the intensely colored ABTS^*∙*+^ cation. In this method, antioxidant capacity was measured by the decolorization ability of some phenolic and flavonoid compounds from reaction of ABTS radicals and the antioxidants added to the medium. The ABTS assay can be applied to both lipophilic and hydrophilic compounds [52]. This method is based on the principle that the ABTS radical cation shows maximum absorbance at 734 nm. Reaction with the ABTS radical occurs in a time as short as 0.25 to 0.5 min. The radical scavenging performance of free radical scavengers can be determined by monitoring the decrease in absorbance at 734 nm [47].

The ABTS^∙+^ radical scavenging activity was determined for concentrations of 5, 10, and 20 *μ*g/mL of reference antioxidants and the phenolic and flavonoid compounds. According to the results, 2,4,6-trihydroxybenzaldehyde, oenin, callistephin, and ID-8 at 5 *μ*g/mL concentration exhibited higher ABTS^∙+^ radical scavenging activities than the other phenolic and flavonoid compounds and the reference antioxidants (BHT, trolox, and *α*-tocopherol) (Table 2). In addition, callistephin and 2,4,6-trihydroxybenzaldehyde with IC_50_ values of 5.28 and 5.54 *μ*g/mL, respectively, showed higher ABTS^∙+^ radical scavenging activity than the phenolic and flavonoid compounds and the reference antioxidants BHT, *α*-tocopherol, and trolox did. In addition, 3,4-dihydroxy-5-methoxybenzoic acid, oenin, silychristin, pelargonin, ID-8, and malvin exhibited IC_50_ values of 6.05, 6.60, 6.71, 6.71, 6.80, and 7.20 (*μ*g/mL), respectively. They showed better ABTS radical scavenging activity than the examined phenolic and flavonoid compounds and the reference antioxidants *α*-tocopherol and trolox. In addition, all compounds examined showed dose-dependent ABTS^∙+^ radical scavenging activity.

Another method that is similar to the ABTS radical scavenging assay is the DMPD radical scavenging method. Tohma and Gulçin proposed this new version of the ABTS test [35]. In this method, the ABTS radical is substituted with the stable DMPD^*∙ ***+**^ radical cation formed from N,N-dimethylphenylenediamine [28, 53]. They reported that DMPD^*∙*+^ radical scavenging activity was more efficient and the test was less expensive than the ABTS^*∙*+^ radical scavenging method. DMPD is converted to colored, stable DMPD^∙+^ radical cation in the presence of oxidants and an acidic medium. The visible spectrum of DMPD^∙+^ radical exhibits maximum absorbance at 505 nm. However, DMPD cannot be used with hydrophobic antioxidants because it dissolves in water only [28]. When hydrophobic antioxidants are used, the sensitivity and reproducibility of the assay drop dramatically [54]. Antioxidant compounds decolorize the solution by donating a hydrogen atom to DMPD radicals [28, 41]. DMPD^*∙*^ radical scavenging activity was assayed for different concentrations (10–30 *μ*g/mL) of reference antioxidants and some phenolic and flavonoid compounds. The results showed that the DMPD^*∙*^ scavenging activities of the reference antioxidants and some phenolic and flavonoid compounds were very similar (Table 2). At 30 *μ*g/mL concentration, 2,4,6-trihydroxy-benzaldehyde exhibited better DMPD scavenging activity than the other phenolic and flavonoid compounds and the reference antioxidants BHA, BHT, and *α*-tocopherol. In addition, callistephin and 2,4,6-trihydroxybenzaldehyde with IC_50_ values of 13.27 and 12.80 *μ*g/mL, respectively, exhibited better DMPD^*∙*+^ radicals removal activity than the other phenolic and flavonoid compounds and the reference antioxidants BHA, BHT, *α*-tocopherol, and trolox did. In addition, all the compounds tested here exhibited dose-dependent DMPD^∙+^ radical scavenging activity.

Antioxidants, which can effectively reduce prooxidants, can also effectively reduce Fe^3+^ to Fe^2+^ [55]. Therefore, the reducing power of a compound provides important information about its antioxidant activity. Reduction ability is one of the most important antioxidant properties of a compound [56]. The three methods used to determine reduction activity in this study measured the reduction of Cu^2+^, Fe^3+^ (using ferrozine reagent), and Fe^3+^ (using the FRAP reagent).

The Fe^3+^-Fe^2^ reduction activity of the reference antioxidants and some phenolic and flavonoid compounds using tripyridyltriazine (TPTZ) was determined by measuring the formation of the blue Fe^2+^-TPTZ complex at a wavelength of 593 nm (FRAP assay).

Fe^3+^-Fe^2^ reduction activities of almost all of the reference antioxidants and phenolic and flavonoid compounds increased proportionally with their concentration (Table 3). 3,4-dihydroxy-5-methoxybenzoic acid exhibited better Fe^3+^-Fe^2^ reduction capacity than the reference antioxidants and the other phenolic and flavonoid compounds examined. In addition, callistephin and oenin showed more effective Fe^3+^-Fe^2^ reduction activity than the reference antioxidants trolox and *α*-tocopherol did. When Fe^3+^-Fe^2^ reduction activities of the reference antioxidants and phenolic and flavonoid compounds were compared at 30 *μ*g/mL using the FRAP reagent, the antioxidant activities observed, from highest to lowest were as follows: 3,4-dihydroxy-5-methoxybenzoic acid > BHT > oenin > BHA > callistephin > trolox > malvin > *α*-tocopherol > pelargonin > 2,4,6-trihydroxybenzaldehyde > arachidonoyl dopamine > silychristin > ID-8.

The CUPRAC assay measuring the reduction of Cu^2+^ to Cu^+^ was described by Gülçin et al. [57]. This method is based on the reduction of Cu^2+^ to Cu^+^ at pH 7 in aqueous ethanol with the combined effect of antioxidants in the presence of neocuproine (2,9-dimethyl-1,10-phenanthrene). The Cu^+^ complex formed by the phenols shows maximum absorbance at 450 nm [58]. This method is suitable for a wide variety of antioxidants, both hydrophilic and hydrophobic substances, because it is low-cost, fast, stable, and selective. Furthermore, the chromogenic CUPRAC redox reaction occurs at physiological pH and is commonly used to compare nonprotein thiol-type antioxidants, such as glutathione, as opposed to the FRAP method, which does not respond to antioxidants containing SH groups [59].

The results obtained with this method showed dose-dependent Cu^2+^ reduction activity for all phenolic and flavonoid compounds. In addition, 30 *μ*g/mL, 2,4,6-trihydroxybenzaldehyde, 3,4-dihydroxy-5-methoxybenzoic acid, malvin, oenin, and callistephin exhibited absorbance values of 0.431, 0.464, 0.456, 0.466, and 0.474, respectively, in the Cu^2+^-Cu^+^ reduction assay, which were higher than the values obtained for other phenolic and flavonoid compounds and trolox and *α*-tocopherol, the reference antioxidants (Table 3).

## 4. Conclusion

Our data demonstrate the difference in antioxidant activities of the reference antioxidants and selected phenolic and flavonoid compounds in different assays. This may be due to the fact that the different antioxidant capacity determining methods have different specificities for different solvents, reagents, pH conditions, or hydrophilic and hydrophobic substances. Furthermore, molecular size and the number and type of functional groups of the phenolic and flavonoid compounds may be important. Oenin, malvin, arachidonoyl dopamine, callistephin, silychristin, and 3,4-dihydroxy-5-methoxybenzoic acid exhibited better antioxidant activities than the reference antioxidants did. Therefore, these compounds may have the potential to protect and maintain food and medicines and reduce oxidative stress or increase antioxidant capacity* in vivo*: this conclusion should be further validated by future studies.

## Figures and Tables

**Table 1:** The comparison of lipid peroxidation inhibition percentages and ferrous ion (Fe^2+^) chelating activities of reference antioxidants and some phenolic and flavonoid compounds (10 *μ*g/mL for chelating activity and 20 *μ*g/mL for lipid peroxidation inhibition).

Antioxidant	Fe^2+^ ions chelating activity with ferrozine reagent		Fe^2+^ ions chelating activity with bipyridyl reagent		Total antioxidant activity
	IC_50_ (*µ*g/mL)	% activity	IC_50_ (*µ*g/mL)	% activity	% inhibition
BHA	32.47	27.34	42.03	24.46	96.39
BHT	30.07	34.89	31.98	28.86	63.63
*α*-Tocopherol	25.73	38.41	12.66	72.53	9.10
Trolox	49.44	21.35	18.56	30.86	93.57
EDTA	—	—	7.32	95.80	—
Malvin	18.54	52.21	14.34	65.73	95.16
ID-8	18.33	54.16	8.80	88.06	97.98
Pelargonin	25.72	39.58	14.30	51.46	93.93
Silychristin	36.78	25.78	24.83	14.20	95.45
Callistephin	34.51	22.73	20.80	33.06	97.58
Oenin	28.95	22.00	26.47	16.40	96.75
Arachidonoyl Dopamine	20.37	50.65	11.08	73.86	51.14
3,4-Dihydroxy-5-methoxybenzoic acid	26.93	36.32	52.37	92.00	17.23
2,4,6-Trihydroxy benzaldehyde	32.16	32.16	17.93	48.00	28.24

The IC_50_ values were calculated by means of metal chelating and total antioxidant activity graphs from values measured at different concentrations (10–30 *µ*g/mL) of reference antioxidants and the phenolic and flavonoid compounds.

**Table 2:** Free radical scavenging activities (%) of reference antioxidants and selected phenolic and flavonoid compounds at 10 *µ*g/mL concentration for ABTS and DMPD and at 20 *µ*g/mL for O_2_^∙−^ and DPPH^*∙*^.

Antioxidant	DPPH^∙^		DMPD^∙+^		ABTS^∙+^		O_2_^∙−^	
	IC_50_ (*µ*g/mL)	% activity	IC_50_ (*µ*g/mL)	% activity	IC_50_ (*µ*g/mL)	% activity	IC_50_ (*µ*g/mL)	% activity
BHA	8.09	98.64	15.34	15.34	3.60	99.80	23.37	41.06
BHT	11.89	96.28	15.26	15.26	6.04	99.80	15.02	64.60
*α*-Tocopherol	17.25	93.85	15.14	64.14	8.47	70.63	23.21	42.00
Trolox	14.13	95.21	13.90	64.57	7.39	83.72	23.21	44.00
Malvin	21.36	92.21	16.47	59.85	7.20	81.72	30.97	33.06
ID-8	536.41	93.85	17.56	60.28	6.80	82.63	34.73	30.66
Pelargonin	67.73	91.50	15.86	65.85	6.71	93.54	14.13	63.40
Silychristin	86.16	91.07	17.62	61.71	6.71	89.18	18.19	51.86
Callistephin	20.64	95.57	12.80	72.71	5.54	99.72	19.70	49.60
Oenin	16.72	95.35	15.46	65.85	6.60	99.72	16.70	56.40
Arachidonoyl dopamine	84.10	98.50	18.02	59.71	12.54	42.00	23.95	39.40
3,4-Dihydroxy-5-methoxybenzoic acid	10.69	98.64	17.80	64.42	6.05	99.80	11.47	73.40
2,4,6-Trihydroxy benzaldehyde	28.86	96.78	17.93	70.00	5.28	99.72	16.54	56.80

The IC_50_ values were calculated by means of radical scavenging activity graphs from the values measured at different concentrations (10–30 *µ*g/mL) of reference antioxidants and some phenolic and flavonoid compounds.

**Table 3:** Fe^3+^ and Cu^2+^ reducing activities of reference antioxidants and selected phenolic and flavonoid compounds at 30 *µ*g/mL concentration.

Antioxidant	FRAP assay (593 nm)	CUPRAC assay (450 nm)
BHA	2.344	0.489
BHT	2.430	0.476
*α*-Tocopherol	2.259	0.403
Trolox	2.086	0.330
Malvin	2.189	0.431
ID-8	0.615	0.145
Pelargonin	2.064	0.385
Silychristin	1.181	0.259
Callistephin	2.328	0.456
Oenin	2.351	0.464
Arachidonoyl Dopamine	1.392	0.308
3,4-Dihydroxy-5-methoxybenzoic acid	2.458	0.474
2,4,6-Trihydroxybenzaldehyde	1.839	0.466
